# Effect of Printing Parameters on Dimensional Error, Surface Roughness and Porosity of FFF Printed Parts with Grid Structure

**DOI:** 10.3390/polym13081213

**Published:** 2021-04-09

**Authors:** Irene Buj-Corral, Ali Bagheri, Maurici Sivatte-Adroer

**Affiliations:** 1 Department of Mechanical Engineering, Barcelona School of Industrial Engineering (ETSEIB), Universitat Politècnica de Catalunya-Barcelona Tech (UPC), 08028 Barcelona, Spain; ali.bagheri@upc.edu; 2 Department of Mechanical Engineering, Vilanova i la Geltrú School of Engineering (EPSEVG), Universitat Politècnica de Catalunya-Barcelona Tech (UPC), 08880 Vilanova i la Geltrú, Spain; maurici.sivatte@upc.edu

**Keywords:** FFF, FDM, roughness, dimensional error, porosity, layer height, speed, temperature, flow rate, multi-objective optimization

## Abstract

Extrusion printing processes allow for manufacturing complex shapes in a relatively cheap way with low-cost machines. The present study analyzes the effect of printing parameters on dimensional error, roughness, and porosity of printed PLA parts obtained with grid structure. Parts are obtained by means of the fused filament fabrication (FFF) process. Four variables are chosen: Layer height, temperature, speed, and flow rate. A two-level full factorial design with a central point is used to define the experimental tests. Dimensional error and porosity are measured with a profile projector, while roughness is measured with a contact roughness meter. Mathematical regression models are found for each response, and multi-objective optimization is carried out by means of the desirability function. Dimensional error and roughness depend mainly on layer height and flow rate, while porosity depends on layer height and printing speed. Multi-objective optimization shows that recommended values for the variables are layer height 0.05 mm, temperature 195 ºC, speed 50 mm/min, and flow rate 0.93, when dimensional error and roughness are to be minimized, and porosity requires a target value of 60%. The present study will help to select appropriate printing parameters for printing porous structures such as those found in prostheses, by means of extrusion processes.

## 1. Introduction

In recent years, the fused filament fabrication (FFF) technique, also known as fused deposition modeling (FDM), has been employed to generate not only prototypes, but also small series of parts [1,2,3]. Furthermore, the number of studies related to this topic is growing continuously [4,5]. The FFF technology has important advantages such as the possibility to obtain complex shapes, even with porous structures, or the possibility to use different plastic materials and metal- or ceramic-filled materials [6]. In the FFF technology, crucial aspects, for instance dimensional and geometric precision or surface quality, still need to be improved in comparison with conventional manufacturing processes such as machining [7].

Extrusion printing process can be used in different biomedical applications, for example in tissue engineering or in drug delivery [8]. Specifically, 3D bioprinting offers the possibility to build complex structures that can be used for tissue regeneration, with bioinks that are used as the biomaterials laden with cells and other biological materials [9]. As for FFF processes, different filaments of pharmaceutical-grade polymers can be used nowadays to manufacture drugs, for example from cellulose derivatives, poly (ethylene oxide), poly (vinyl alcohol), etc. The filaments can also contain active pharmaceutical ingredients (APIs), although most of them are still not commercially available at pharmaceutical grade [10,11]. Other medical applications of the extrusion processes are the fabrication of surgical guides, dental fixtures, and customized patient specific implants [12,13]. For example, FFF patterns are currently being used to manufacture implants by means of investment casting [14]. Some implants have also directly been printed by means of FFF, including craniofacial reconstruction and orthopedic spacers in polymethylmethacrylate (PMMA) [15].

One of the main advantages of manufacturing prostheses with 3D printing processes is the possibility to obtain patient specific parts. For this reason, it is advisable to achieve good dimensional accuracy. If low-cost machines are to be used in order to make the technology affordable, accuracy will depend greatly on the printing parameters selected. Some authors have studied the dimensional accuracy of FDM printed parts. For example, Caminero et al. [16] found that the best dimensional accuracy for polylactic acid (PLA) and PLA-graphene parts was in the *Z*-axis, when printed in flat and on-edge orientations. Beniak et al. [17] assessed the dimensional accuracy of FDM printed parts as a function of layer height and temperature. They found that the worst accuracy corresponded to high layer height and high printing temperature. Nancharaiah et al. [18] studied the surface roughness and dimensional accuracy of FDM printed parts in ABS. According to their work, dimensional accuracy worsened for high layer height of 0.33 mm and improved for high raser angle of 45º. Pennington et al. [19] observed that dimensional accuracy depended on the part size, its location in the work envelope, and the envelope temperature in FDM printing. Garg et al. [20] did not find a significant influence of a cold vapor treatment on the dimensional accuracy of ABS parts.

In prostheses, roughness is to be minimized on those surfaces that will be in contact with other parts. For example, in hip prostheses, the internal surface requires good surface finish in order to reduce friction with the femoral head [21]. In extrusion printing processes, roughness depends on the measuring direction. A certain roughness level on the lateral walls of specimens is inherent to the process, because of the superposition of layers [22,23]. Specifically, a study by Alsoufi et al. [24] indicated that the measuring direction of 90° gives the most representative value of Ra distribution than other angles (0° and 45°). Different parameters influence surface roughness to be obtained. For example, Luzanin et al. [25] used a 2^2^ design and showed that the extrusion speed had a dominant, statistically significant effect on Ra, while the extrusion temperature and their interaction were not seen to be significant. Galantucci et al. [26] tested the influence of different parameters on Ra in FDM-built ABS (acrylonitrile butadiene styrene) parts employing factorial analysis. They observed that high tip diameter, low raster width, and low slice height led to lower roughness. Rahman et al. [27] studied the influence of bed temperature, nozzle temperature, printing speed, infill, layer thickness, and the number shells of the parts on surface roughness and dimensional deviations of ABS parts. The best results were achieved with high bed temperature, medium nozzle temperature, low print speed, medium infill, low layer thickness, and high number of shells. Sajan et al. [28] used the Taguchi method to evaluate surface roughness. Their analysis showed that main parameters influencing surface texture and circularity error were bed temperature, number of loops, nozzle temperature, print speed, layer thickness and infill, in ABS parts. Hartcher-O’Brien et al. [29] studied the effect of print speed, angle and layer height on roughness parameters Ra and Rq. They observed that surface roughness increased with layer height and decreased with print speed and angle.

Porosity and pore size of printed scaffolds, for example those used in prostheses, are important, because they are related to cell growth as well as to nutrient transport [30]. Moreover, although higher scaffold surface area is known to increase the tissue volume, some studies conclude that the surfaces should also be concave to favor cell growth [31]. Besides, the rough surfaces have an important effect on the transport of fluids through porous media [32]. The usual requirements for prostheses are: Pore size between 100 μm and 500 μm, and total porosity between 40% and 80% [33,34]. Montazerian et al. studied the influence of pore size and porosity of printed scaffolds on their permeability [35]. Regarding measurement of porosity, it is possible to determine pore size from its three-dimensional image obtained by means of computed tomography [36]. This technique has been employed, for instance, to determine the porosity of the grid structure in FFF processes [37]. However, few studies have addressed the effect of FFF printing parameters on the porosity of printed parts.

The main aim of the present paper is to study and analyze the influence of the FFF printing parameters on surface roughness, dimensional error, and porosity of porous structures. In addition, the selection of optimal printing parameters in order to simultaneously minimize dimensional error and roughness of customized parts such as prostheses, as well as to achieve a target porosity value to favor cell growth, is investigated. On the other hand, an alternative way to measure porosity is presented here for regular structures such as the grid one, in which the volume of pores is obtained by measurement of the pore dimension with a profile projector. This methodology can be applied to porous structures of different materials, provided that they have a regular structure with through holes.

## 2. Materials and Methods

### 2.1. Methods

#### 2.1.1. Printing Process

PLA filament of 2.85 mm in diameter was used as a feedstock material. Experiments were performed using a Sigma R17 from BCN 3D Technologies. The maximum print volume is 210 × 297 × 210 mm. It has a resolution of 12.5 µm on the X and Y axes and a resolution of 1 µm on the *Z*-axis. Maximum heating temperature is 280 °C for the printing head and 100 °C for the printing bed. The nozzle diameter was 0.4 mm, and three shells were defined around the parts, corresponding to a total thickness of 1.2 mm.

Four variables were chosen in this work: Layer height, temperature, speed, and flow-rate. Layer height is the difference between one layer and the next deposited layer. As a general trend, roughness and dimensional error increase with layer height in FFF processes [18]. The extrusion temperature is the temperature that is required to melt and extrude the material and later deposit it. A temperature value should be chosen in which the melted material is not damaged. According to some authors, an increase in printing temperature worsens the dimensional accuracy of the parts [17], while other authors did not find a significant effect of temperature on roughness [24]. The print speed refers to the velocity at which the printer head moves when printing the parts. Increasing the speed can cause vibrations and errors in the printing process, which decrease the quality of the printed specimens. However, print speed has usually a lower effect on surface roughness than other parameters such as layer height [38]. The flow rate parameter is also known as the extrusion multiplier. It represents the amount of material that comes out of the extruder compared to the theoretical one. Its effect on surface finish and dimensional error has been scarcely studied in the literature about FFF processes. In a different extrusion printing process, direct ink writing (DIW) of ceramic inks, the flow rate or extrusion multiplier parameter had a low effect on surface roughness and dimensional error [39]. However, the flow rate required in FFF or in DIW processes could be quite different because of the different rheological behavior of both materials.

**Figure 1:**
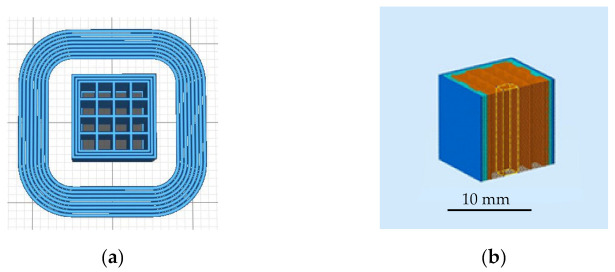
Geometry of the specimens: (**a**) 2D drawing of the grid structure surrounded by a printing skirt, (**b**) 3D drawing of the grid structure with a prismatic channel highlighted in yellow.

In the present study, the samples were designed with a computer-aided program, Solidworks, as cubes with a size of 10 × 10 × 10 mm, with the grid structure and an infill ratio of 40%. The structure has 16 void prismatic channels with square bases (Figure 1a,b).

A 2-level full factorial design with 4 variables (2^4^) was defined with the help of Minitab 19. Three central points were added to the design, with a total number of 19 experiments. The employed levels for the variables are shown in Table 1.

In this study, the layer height range was chosen between 0.05 mm and 0.25 mm. The value of 0.05 mm is the minimum value that could be printed in PLA in this case, and the upper value of 0.25 mm is the highest recommended value, corresponding to 80% of the nozzle diameter. Temperature was selected between 190 and 210 °C, as it is usually recommended for PLA, making it possible to melt the material correctly and the layers adhere to the contiguous ones. Printing speed varied between 30 mm/s and 50 mm/s, as is usual for PLA. Finally, for the flow rate some preliminary tests were carried out, where the initial values were adjusted between 0.93 and 0.97.

The considered responses were: Dimensional error, roughness parameter Ra (arithmetical mean deviation of the profile), and porosity.

#### 2.1.2. Dimensional Error Measurement

In the present work, dimensional error is defined as the relative difference between the theoretical and the measured dimensions of the printed parts. The theoretical piece corresponds to a cube of 10 × 10 × 10 mm. In this case, the dimensional error in width is considered. To do this, the width of the four lateral walls of the cubes were measured with a Mitutoyo PJ300 profile projector. Then, the relative error was calculated and the average value of the four errors was calculated for each part.

#### 2.1.3. Roughness Measurement

Roughness was measured with a Taylor Hobson Talysurf 2 contact roughness meter. One measurement was performed on the lateral wall of each sample.

**Figure 2:**
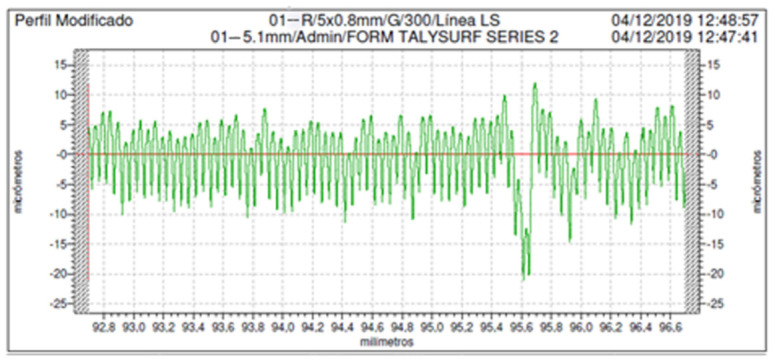
Example of a roughness profile of experiment 1, measured on a lateral wall of the specimens.

Figure 2 shows an example of a measured roughness profile (experiment 1), with round peaks and sharp valleys, that is typical of the FFF printed parts.

The profile is quite regular, suggesting that the different material layers were correctly deposited. In general, the peak width corresponds to the layer height of 0.05 mm that was employed in this experiment.

#### 2.1.4. Porosity Measurement

The porosity or void fraction is the ratio of the volume of voids within a structure over the total volume. It can take values between 0 and 1, or as a percentage between 0% and 100%.

In the present work, a regular structure was considered with the grid pattern and 40% infill ratio. This leads to 16 void channels with square cross-section (Figure 1a). The volume of each pore is calculated by multiplying the dimensions of the sides (1.5 mm) times the dimensions of the side (1.5 mm) times the length of the channels (10 mm). The theoretical porosity of the structure is 60%, corresponding to an infill rate value of 40%.

The profile projector was used to measure the porosity of the specimens. To do this, the dimensions of the four central holes of the structure, which are not influenced by the shell of the parts, as well as the length of the parts were measured and the volume of the holes was calculated. Then the volume of the holes was compared to the total volume of the part (without considering the shells).

#### 2.1.5. Regression Models and Multi-Objective Optimization

Linear regression models were searched for each response with Minitab 19. Once the models for each of the responses were obtained, multi-objective optimization was performed. It simultaneously seeks to minimize dimensional error and roughness, while maintaining a target porosity value of 60%, which is the theoretical porosity value for an infill ratio of 40%.

Multi-objective optimization is a useful approach in FFF processes, in order to select the optimal combination of printing parameters [40]. In the present work, the desirability function method is employed for this purpose. A desirability function is a piecewise objective function that ranges from 0 outside of the limits to 1 when it reaches the goal. By means of numerical optimization, a point is found that maximizes the desirability function. The desirability function can be defined for either a single response or for different responses simultaneously. The overall desirability is the geometric mean of the desirability functions for the different responses considered [41].

## 3. Results and Discussion

### 3.1. Measured Values

Table 2 shows the results for dimensional error, roughness, and porosity, as well as the printing conditions of the different experiments.

For the central point (experiment 17), the test was repeated three times. Mean values and standard deviation values for the responses are presented in Table 3.

Highest dimensional error was found for experiments 1 and 15, suggesting that the low layer height value of 0.05 mm provides some incorrectly printed parts in this case. Lowest dimensional error was observed for experiments 2, 5, and 6, which have in common low temperature and low flow rate, which avoid an excessive fluidity of the material. These results are in accordance with the work of Beniak et al. [17], who recommend low temperature in order to reduce dimensional error. To sum up, within a certain layer height range that ensures correct printing, low temperature, and low flow rate are recommended in order to avoid an excess of material that would increase the dimensional error.

Highest roughness values above 18 µm correspond to experiments 4 and 8, obtained with high layer height, high temperature, and low speed. Lowest roughness values were reported in experiments 3 and 5 and 13, printed with low layer height. It is well known that roughness in the lateral walls of FDM printed parts increases with layer height [22,23]. Although a low layer height of 0.05 mm would be recommended in order to minimize roughness, the rest of the variables should be selected carefully so as to avoid excessive dimensional error, as was explained in the previous paragraph.

In the present work, the target porosity value of 60% ± 1% is achieved under different combinations of parameters, for example in experiments 3, 9, 11 and 13, all of them printed with low layer height.

### 3.2. Regression Model for Dimensional Error

The regression equation for dimensional error in uncoded units is presented in Equation (1). The R-sq (adj) parameter is 87.17%.

(1) $$\mathrm{Dim}.\;\mathrm{Error}\;\left(\%\right)=99.5-424.1\;LH-0.0673\;TE-2.751\;PS-87.1\;FR\;+10.24\;LH\cdot PS+437.5\;LH\cdot FR+0.002225\;TE\cdot PS\;+2.394\;PS\cdot FR-10.63\;LH\cdot PS\cdot FR-0.526\;CtPt$$

**Figure 3:**
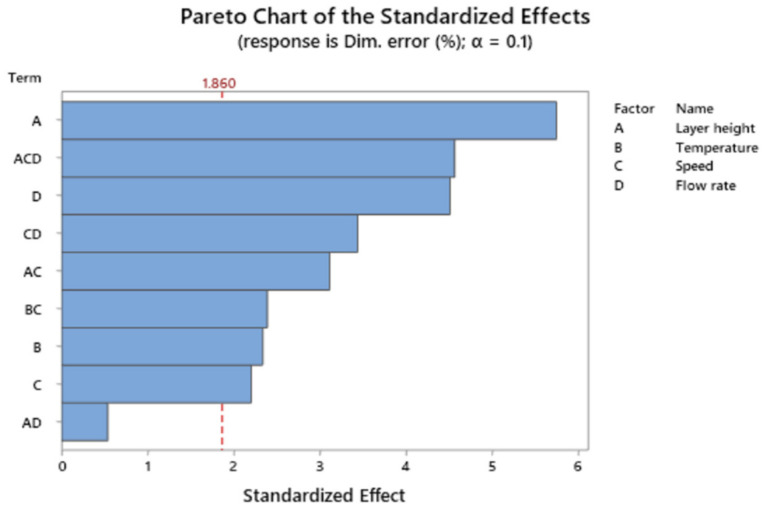
Pareto Chart of the standardized effects for the response dimensional error.

Figure 3 depicts the Pareto chart of the standardized effects for dimensional error.

Main terms influencing dimensional error are layer height followed by the interaction of layer height, print speed, and flow rate, and by flow rate.

**Figure 4:**
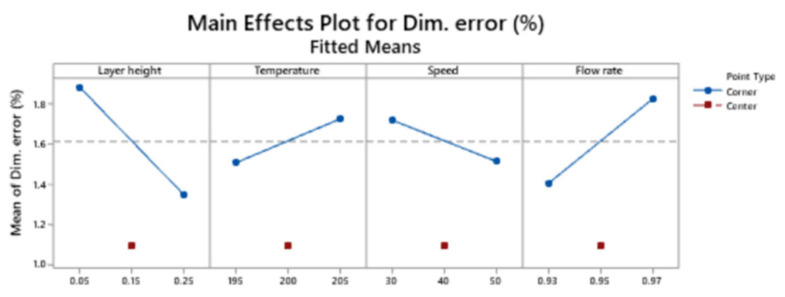
Pareto chart of the standardized effects for the response dimensional error.

Figure 4 shows the main effects plot for dimensional error. It reveals that curvature is significant in this model. The lowest dimensional error is obtained with the medium value for all the variables.

**Figure 5:**
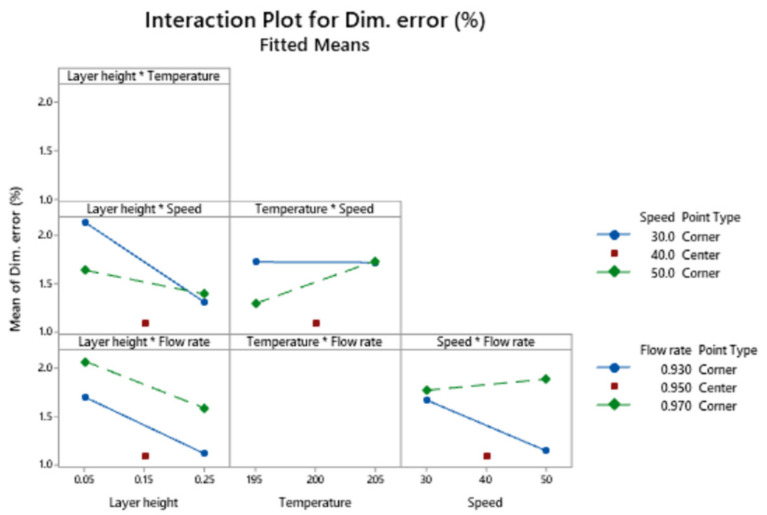
Interaction plot for the response dimensional error.

Figure 5 corresponds to the interaction plot for dimensional error.

The interaction between speed and flow rate shows that low dimensional error is obtained when the combination of high speed and low flow rate is selected. The interaction between layer height and speed indicates that low dimensional error is obtained with high layer height regardless of speed.

As observed by Beniak et al. [17], dimensional is directly related to temperature, since higher temperature means easier material flow. Nancharaiah et al. [19] found stable dimensional error values between layer height 0.17 and layer height 0.25 mm, while error increased when layer height of 0.33 mm was employed. In the present research, as a general trend, dimensional error is higher for layer height 0.05 mm than for layer height 0.25 mm. This suggests that extreme layer height values of either 0.05 mm or 0.33 mm could worsen the dimensional accuracy. In the present work, dimensional error values of up to 2.6% are obtained. Messimer et al. [42] reported slightly higher values of up to 3% for high-temperature polylactic acid (HTPLA).

### 3.3. Regression Model for Roughness

The regression model for roughness in uncoded units is presented in Equation (2). The R-sq (adj) coefficient is 92.22%.

Ra (um) = 114.5 + 493 LH − 0.643 TE − 3.12 SP + 21.2 FR − 466 LH·FR + 0.01531 TE·SP − 4.060 Ct Pt(2)

**Figure 6:**
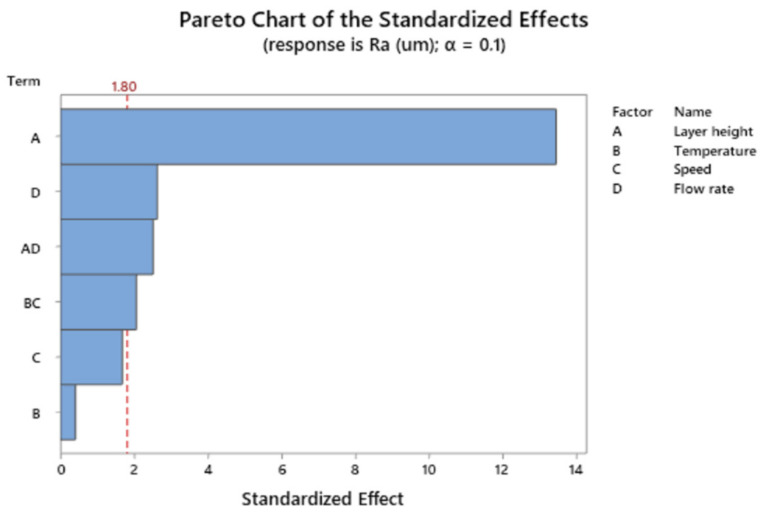
Pareto chart of the standardized effects for the response Ra.

Figure 6 depicts the Pareto Chat of the standardized effects for Ra.

The main term influencing roughness is layer height, followed by flow rate and by the interaction between layer height and flow rate.

**Figure 7:**
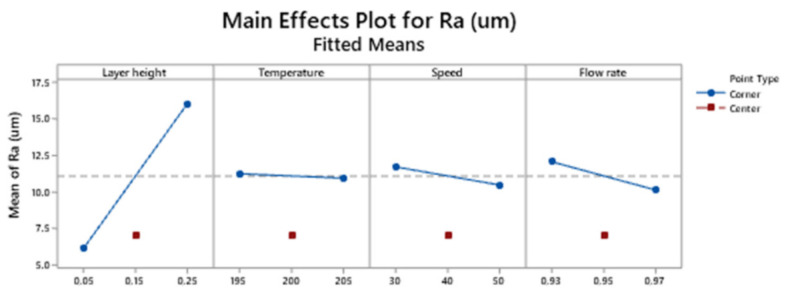
Main effects plot for the response Ra.

Figure 7 shows the main effects plot for roughness parameter Ra.

Lowest roughness value is achieved with low layer height, when the rest of the variables are kept in their medium values. Curvature is significant in this model.

**Figure 8:**
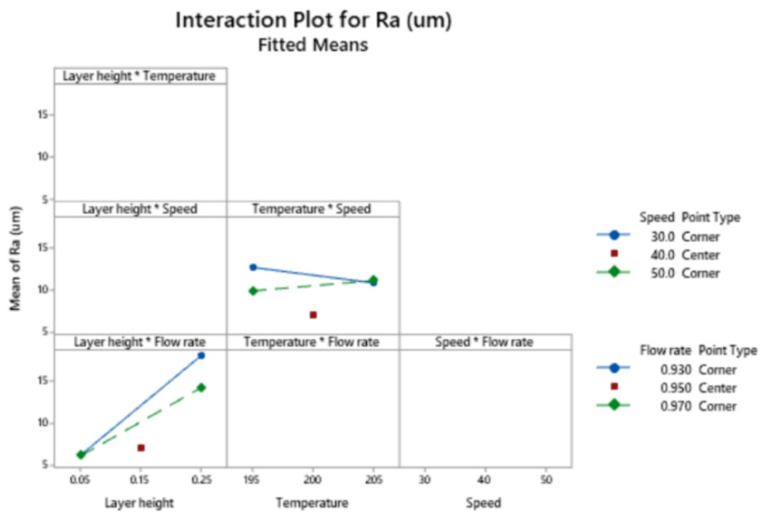
Interaction plot for the response Ra.

Figure 8 depicts the interaction plot for Ra.

The interaction between layer height and flow rate shows that the lowest roughness corresponds to low layer height, regardless of the flow rate employed.

The great influence of layer height on surface roughness of FDM printed parts has been reported by different authors [22,26,27]. In the present work, the lowest Ra value achieved in the lateral walls of the parts that were considered in the present research was around 3.9 µm when low layer height, low temperature and a high speed are selected. When layer height of 0.25 mm is used, Ra values up to almost 19 µm are found, which are similar to those obtained in the lateral walls of cylindrical shapes for the same layer height [22].

### 3.4. Regression Model for Porosity

The regression equation for porosity in uncoded units is presented in Equation (3). The R-sq (adj) coefficient is 68.06%. Curvature is significant in this model.

Porosity (%) = −28.6 − 30.9 LH + 0.318 TE − 0.031 PS + 25.7 FR + 1.479 LH·PS − 7.25 Ct Pt(3)

**Figure 9:**
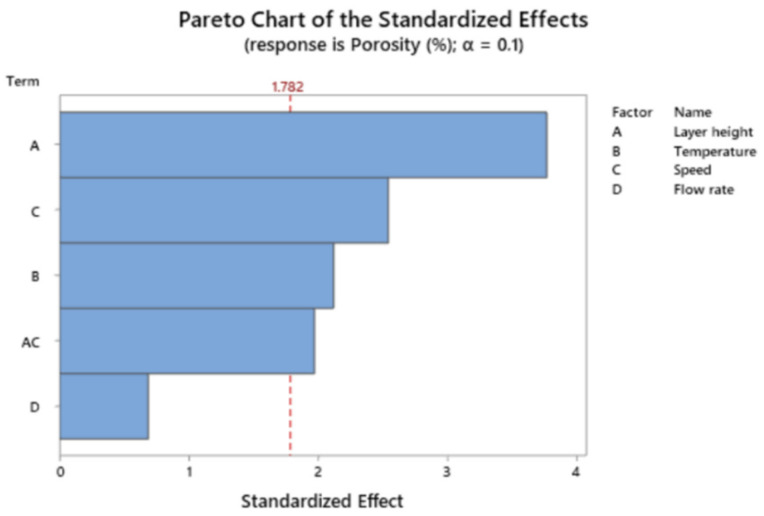
Pareto chart of the standardized effects for the response porosity.

Figure 9 shows the Pareto chart of the standardized effects for porosity.

The main terms influencing porosity are layer height, followed by speed and temperature.

**Figure 10:**
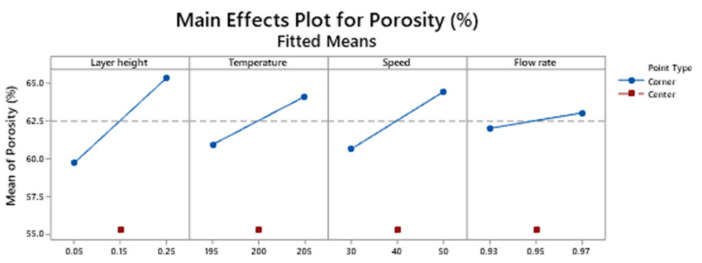
Main effects plot for the response porosity.

Figure 10 depicts the main effects plot for the response porosity.

The target value of 60 % for porosity is obtained, as a general trend, when low layer height is selected.

**Figure 11:**
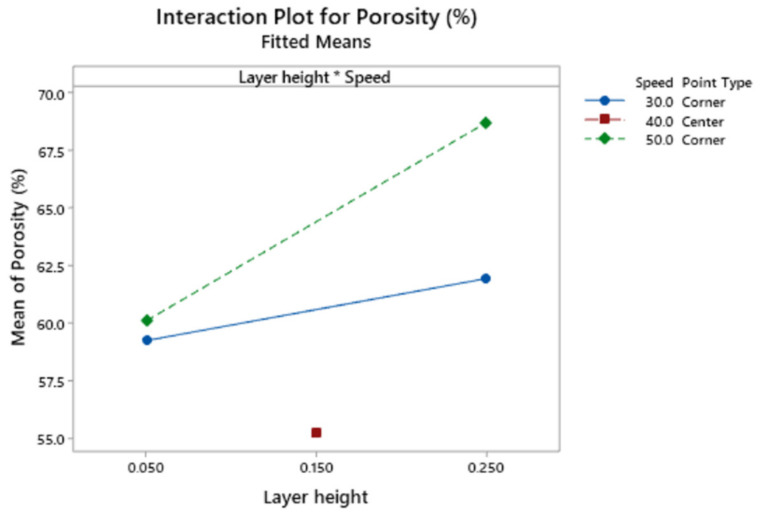
Interaction plot for the response porosity.

Figure 11 corresponds to the interaction plot for the response porosity.

The interaction plot in Figure 11 shows that the target porosity value is obtained with low layer height, regardless of speed. If high layer height were selected, then low print speed would be recommended.

Porosity values obtained range between 53.76% and 72.33%, with 60% being the target. According to a previous work, the measured porosity of the grid structures can be lower than the theoretical porosity because of because of an excess of material [37]. In the present work, higher porosity than expected was also observed in some cases, with thin-walled structures. The structures corresponding to the target porosity of 60 % showed regular structures.

### 3.5. Multi-Objective Optimization

Table 4 contains the parameters used for the multi-objective optimization with the desirability function method, and Table 5 shows the solution of the optimization. The weight was 1 in all cases, corresponding to the use of a linear function to define the desirability of the responses. The three responses were given the same importance value of 1.

Table 5 shows that, when all the responses are given the same importance, it is recommended to select low layer height, low temperature, high speed and low flow rate. The compound desirability is 0.852.

**Figure 12:**
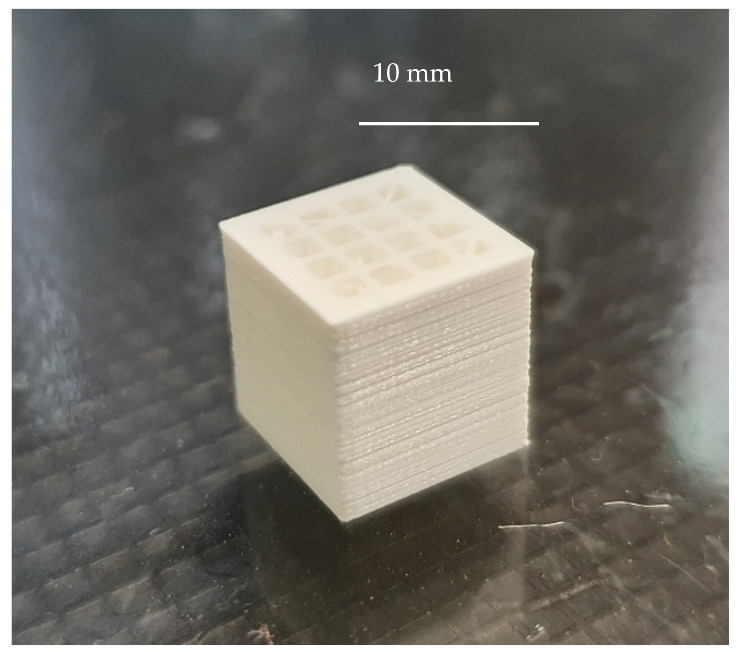
Photograph of a sample that was printed using the optimized values for the variables, when all the responses are given the same importance.

Figure 12 shows a specimen that was printed using the optimized values for the variables, presented in Table 5.

The deposition of the material layer upon layer is correct, and the 16 holes show a regular structure with homogeneous wall thickness among then.

## 4. Conclusions

In the present work, cubic parts are printed in PLA by means of FDM, using a grid structure with 40% infill. Different experiments are performed, in which layer height printing temperature, print speed, and flow rate are varied. The main conclusions of the paper are as follows:

- Dimensional error values between 0.82% and 2.60% were obtained. As a general trend, selecting layer height of 0.25 mm provides lower dimensional error than layer height of 0.05 mm, suggesting that the latest could be too low to assure low dimensional error under certain printing conditions. Low flow rate is also recommended in order to minimize dimensional error.
- Ra roughness values between 3.9 µm and 18.8 µm were obtained. The lower the layer height, the lower roughness is.
- Porosity values range between 53.76% and 72.33%, with a target porosity value of 60%. As a general trend, the target porosity value is achieved with low layer height.
- Dimensional error and surface roughness Ra depend mainly on layer height and flow rate. Porosity is mainly influenced by layer height and print speed.
- According to multi-objective optimization, it is recommended to select low layer height, low temperature, high print speed and low flow rate in order to simultaneously minimize dimensional error and roughness and to obtain the target porosity value.

The present work will help to select most appropriate printing parameters to print porous structures with the grid pattern, in extrusion processes. For example, the results will be used in future works to manufacture plastic prototypes for hip prostheses, with the shape of a hemispherical cup and a porous external layer that will favor its fixation by means of osseointegration.

## Figures and Tables

**Table 1:** Ranges for the variables.

Variable	Acronym	Low Level	High Level
Layer height (mm)	LH	0.05	0.25
Temperature (°C)	TE	195	205
Print speed (mm/s)	PS	30	50
Flow rate (%)	FR	0.93	0.97

**Table 2:** Printing conditions and results for porosity, roughness, and dimensional error for the 19 experiments.

Experiment	LH (mm)	TE (°C)	PS (mm/s)	FR (%)	Dimensionl Error (%)	Ra (μm)	Porosity (%)
1	0.05	195	30	0.93	2.35	8.342	58.99
2	0.25	195	30	0.93	1.00	17.713	53.76
3	0.05	205	30	0.93	2.28	5.074	59.07
4	0.25	205	30	0.93	1.03	18.826	64.92
5	0.05	195	50	0.93	1.01	3.875	58.13
6	0.25	195	50	0.93	1.01	17.270	66.61
7	0.05	205	50	0.93	1.14	7.230	64.94
8	0.25	205	50	0.93	1.40	18.082	55.82
9	0.05	195	30	0.97	2.03	6.190	59.52
10	0.25	195	30	0.97	1.49	18.233	63.52
11	0.05	205	30	0.97	1.85	7.053	59.38
12	0.25	205	30	0.97	1.69	12.179	65.54
13	0.05	195	50	0.97	1.78	5.140	60.23
14	0.25	195	50	0.97	1.36	13.082	66.46
15	0.05	205	50	0.97	2.60	5.804	57.07
16	0.25	205	50	0.97	1.78	13.149	72.33
17-1	0.15	200	40	0.95	0.82	8.150	54.41
17-2	0.15	200	40	0.95	1.29	6.580	56.11
17-3	0.15	200	40	0.95	1.15	6.322	55.20

**Table 3:** Mean and standard deviation values for dimensional error, Ra roughness, and porosity, for experiment 17.

Response	Mean	Standard Deviation
Dimensional error (%)	1.09	0.24
Ra (µm)	7.018	0.989
Porosity (%)	55.24	0.85

**Table 4:** Parameters of the multi-objective optimization by means of the desirability function method.

Response	Goal	Lower	Target	Upper	Weight	Importance
Dim. error (%)	Minimum		0.820	2.600	1	1
Ra (um)	Minimum		3.875	18.826	1	1
Porosity (%)	Target	53.76	60.00	72.33	1	1

**Table 5:** Solution of the multi-objective optimization by means of the desirability function method.

LH	TE	PS	FR	Dimensional Error (%)	Ra (µm)	Porosity (%)	Composite Desirability
0.05	195	50	0.93	0.86	4.894	57.99	0.852

## Data Availability

Not applicable.
